# Effect of platelet-rich plasma combined with simvastatin in the treatment of steroid-induced avascular necrosis of the femoral head

**DOI:** 10.12669/pjms.41.5.11518

**Published:** 2025-05

**Authors:** Yanchen Chu, Chun Rong, Huili Qu, Haining Zhang, Yuan Fang

**Affiliations:** 1 Yanchen Chu, Department of Orthopedics, The Affiliated Hospital of Qingdao University, 59 Haier Road, Qingdao, Shandong Province 266000, P.R. China; 2 Chun Rong, Department of Orthopedics, The Affiliated Hospital of Qingdao University, 59 Haier Road, Qingdao, Shandong Province 266000, P.R. China; 3 Huili Qu, Department of Orthopedics, The Affiliated Hospital of Qingdao University, 59 Haier Road, Qingdao, Shandong Province 266000, P.R. China; 4 Haining Zhang, Department of Orthopedics, The Affiliated Hospital of Qingdao University, 59 Haier Road, Qingdao, Shandong Province 266000, P.R. China; 5 Yuan Fang, Department of Orthopedics, The Affiliated Hospital of Qingdao University, 59 Haier Road, Qingdao, Shandong Province 266000, P.R. China

**Keywords:** Platelet rich plasma, Simvastatin, Steroid-induced avascular necrosis of the femoral head, Therapeutic effect

## Abstract

**Background & Objective:**

Steroid-induced avascular necrosis of the femoral head (SANFH) is a serious complication of the overuse of glucocorticoids. Platelet-rich plasma (PRP) is a regenerative technique that has shown to effectively repair damaged blood vessels, promote angiogenesis, restore normal blood supply, and promote osteogenesis of the femoral head. This study aimed to analyze the effect of PRP combined with a commonly used simvastatin in treating SANFH.

**Methods:**

In this retrospective single-center study clinical data of all SANFH patients who received simvastatin or PRP combined with simvastatin in The Affiliated Hospital of Qingdao University from June 2022 to April 2024 were retrospectively reviewed. Baseline characteristics, recovery of joint function, treatment effect, and incidence of adverse reactions (ARs) were analyzed.

**Results:**

Clinical data of 146 patients who received simvastatin alone (n=75) or PRP combined with simvastatin (n=71) under the guidance of the treating physician were included in the analysis. After the treatment, the recovery of joint function and the rate of excellent and good therapeutic effect in patients who received PRP combined with simvastatin were significantly higher than in patients who received simvastatin treatment alone (*P*<0.05). Combined treatment was associated with significantly lower levels of total procollagen Type-I N-terminal propeptide (T-PINP), N-terminal molecular fragment (N-MID), and β-isomerized C-terminal telopeptide of Type-I collagen (β-CTX), and higher post-treatment levels of 25 hydroxyvitamin D (25-(OH)-D) compared to simvastatin alone (*P*<0.05). No significant difference was found in the incidence of ARs between the two groups (*P*>0.05).

**Conclusions:**

PRP combined with simvastatin can more effectively restore joint function, regulate bone metabolism state, and improve treatment efficiency in patients with SANFH.

## INTRODUCTION

Avascular necrosis of the femoral head (ANFH) is caused by factors such as trauma, excessive alcohol, and hormone use.^1^,^2^ Research has shown that long-term excessive use of corticosteroids can cause changes in local vascular permeability and abnormal secretion of vasoactive substances in the femoral head, leading to microthrombus formation, damage to vascular endothelial cells, reduced blood flow, and eventually, local damage to normal bone tissue structures and development of steroid-induced avascular necrosis of the femoral head (SANFH).^3^,^4^

SANFH patients often have varying degrees of difficulty in walking and hip pain, which significantly affects their daily lives and activities. Therefore, effective treatment should be implemented for patients in the early stages of the disease.^5^,^6^ However, currently, there is a lack of efficient preventive and curative treatment of SANFH. In early stages, SANFH is often treated by lipid-lowering agents, anticoagulants, vasoactive substances, statins, such as simvastatin, and bisphosphonates.^7^ Simvastatin is commonly used to prevent the development of SANFH as it can regulate endothelial cell function, inhibit smooth muscle cell proliferation, and has antioxidant and anti-inflammatory effects.^7^

Moreover, simvastatin can promote the mobilization, migration, and differentiation of endothelial progenitor cells, improve their function, and accelerate vascular regeneration in ischemic areas.^8^,^9^ Platelet-rich plasma (PRP) is a regenerative technique based on concentrating platelets by centrifuging autologous blood. In addition to platelets, PRP injection into the joint cavity delivers various growth factors that can regulate the damaged local microcirculation environment and facilitate the repair of bone injury, cartilage degeneration, and other conditions.^10^,^11^ A systematic review by Han et al.^12^ recommended PRP injection as an adjunctive therapy for ANFH. However, evidence on the efficacy and safety of PRP combined with simvastatin in SANFH is scarce. This study aimed to analyze the safety and the intervention effect of PRP combined with simvastatin in patients with SANFH. Our results may contribute to developing more effective ways of treating this disease.

## METHODS

This retrospective single-center study included medical records of all SANFH patients who received simvastatin or PRP combined with simvastatin treatment at The Affiliated Hospital of Qingdao University from June 2022 to April 2024.

### Ethical Approval:

Due to the retrospective nature of the study, informed consent was not required. The study was approved by our ethics committee (ethics number: QYFYWZLL29058).

### Inclusion criteria:

- Meet the SANFH diagnostic criteria.^4^
- Hip joint pain, limited internal rotation.
- X-ray examination shows that the collapse of the femoral head is ≥ 50%.
- History of corticosteroid use.
- CT examination showed bone necrosis on the anterior lateral side of the femoral head, while MRI examination suggested the presence of linear features.
- Complete clinical data.

### Exclusion criteria:

- Necrosis caused by other factors leading to femoral head injury.
- Osteoarthritis and bone tumors.
- Significant organ dysfunction.
- Breastfeeding and pregnant women.
- Patients with specific arthritis and rheumatic diseases.
- A history of hip surgery or trauma.

Simvastatin (manufacturer: China National Pharmaceutical Group Shantou Jinshi Pharmaceutical Co., Ltd.; Shantou, China) was taken orally at a dose of 20 mg/time, once a day, for four weeks.

### PRP treatment:

For the PRP preparation, 40 ml of peripheral blood was collected into a 50-ml tube with 3.8% sodium citrate as anticoagulant (final concentration of 0.475% sodium citrate) and centrifuged at 2000×g for 15 min at 22 ± 2ºC. Plasma was collected; the patient was guided to lie flat. Vascular and neural structures together with the injection targets were clarified through ultrasound examination (Philips iU22, Germany). Five ml PRP were injected into the joint cavity using ultrasound guidance. The treatment was administered once a week for a total of four weeks.

### The following data were collected:

- Basic characteristics of patients.
- Recovery of joint function assessed using the Harris hip score (HSS) scale. HSS scale scores pain (44 points), function (47 points), deformity (four points), and joint activity (five points), with a total score of 100. Higher scores indicate better recovery of hip function.^13^
- Therapeutic effect, divided into four levels according to the HSS score: poor (≤ 69), general (70-79), good (80-89), and excellent (≥ 90). Rate of excellent and good = (number of excellent + number of good)/total number×100%.
- Changes in bone metabolism indexes. Fasting venous blood of 5mL was collected from both groups and centrifuged to separate serum. Serum levels of total procollagen Type-I N-terminal propeptide (T-PINP), N-terminal molecular fragment (N-MID), β-isomerized C-terminal telopeptide of Type-I collagen (β-CTX), and 25 hydroxyvitamin D (25-(OH)-D) were measured using Roche Cobas e 601 electrochemiluminescence automatic immunoassay system (Germany).
- Adverse reactions, including abdominal pain, abdominal distension, vomiting and nausea, and headache.

### Statistical analysis:

Data were analyzed using SPSS 25.0 (IBM Corp, NY, USA). The normality of the data was assessed using the Shapiro-Wilk test. Normally distributed data were presented as mean ± standard deviation (SD), and the student t-test was used for comparison between groups. Non-normally distributed data were expressed as median and interquartile intervals, and comparison between groups was assessed using the Whitney *U* test. Counting data were shown as n (%) and compared using the Chi-square test. P<0.05 indicated a statistically significant difference. PRISM 8.0 (GraphPad, San Diego, USA) was used to draw the histogram of HHS score and bone metabolism index changes before and after treatment.

## RESULTS

A total of 146 patients (157 hips) were eligible for this study. The cohort included 66 males and 80 females, aged between 26 and 73 years with a mean of 48.28 ± 8.31 years. There were 78 cases of left hip SANFH, 57 cases of right hip SANFH, and 11 cases of double hip SANFH. Seventy-one patients (77 hips) received PRP combined with simvastatin, and 75 patients (80 hips) received simvastatin alone (Table-I). No significant difference was found in the basic characteristics of both groups (*P*>0.05).

**Table-I:** Comparison of basic characteristics between the two groups.

Item	PRP & simvastatin (n=71)	Simvastatin (n=75)	χ^2^/t/Z	P
Male (yes), n (%)	36 (50.7)	30 (40.0)	1.687	0.194
Age (years), mean±SD	47.75±7.83	48.79±8.76	-0.755	0.452
Affected side, n (%)				
Left side	35 (49.3)	43 (57.3)	0.960	0.619
Right side	30 (42.2)	27 (36.0)		
Both sides	6 (8.5)	5 (6.7)		
Course of disease (months), M(P25/P75)	12 (9-15)	12 (9-16)	-0.603	0.546
BMI (kg/m^2^), mean±SD	22.86±3.00	23.38±3.40	-0.982	0.328
Hyperlipidemia (yes), n (%)	13 (18.3)	10 (13.3)	0.681	0.409
Diabetes (yes), n (%)	11 (15.5)	5 (6.7)	2.912	0.088
Hypertension (yes), n (%)	12 (16.9)	11 (14.7)	0.137	0.711

**Fig.1:**
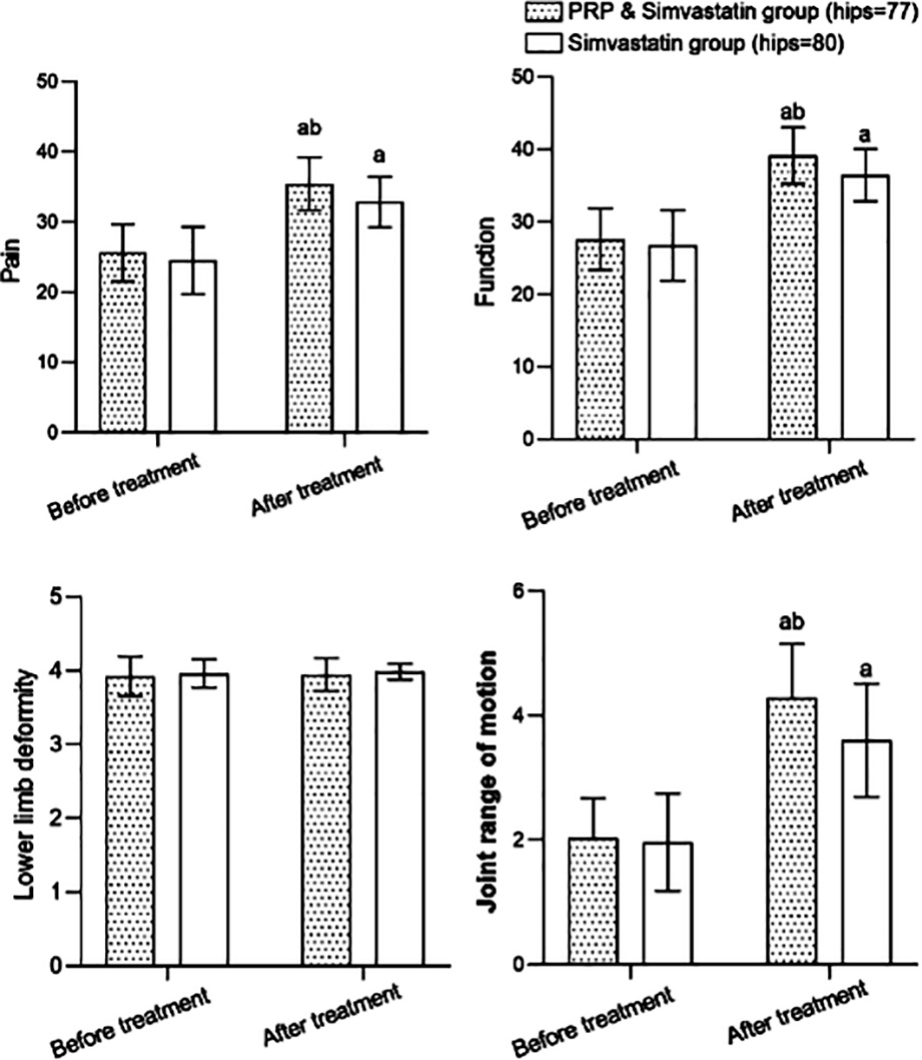
Histogram of changes in HHS scores of the two groups before and after treatment; Compared with before treatment in the same group. ^a^P<0.05; compared with simvastatin group, ^b^P<0.05; HHS: Harris hip score; PRP: Platelet-rich plasma.

Before treatment, no significant difference was found in the HHS scores between the groups (*P*>0.05). After treatment, the scores of pain, function, and joint range of motion were higher in both groups compared to pre-treatment, and the scores in patients who received combined treatment were significantly higher compared to patients who were treated with simvastatin alone (*P*<0.05) (Fig.1). As shown in Table-II, the rate of excellent and good treatment effects of patients in the PRP combined with the simvastatin group (85.7%) was higher than that of the simvastatin group (71.3%) (*P*<0.05).

**Table-II:** Comparison of the excellent and good treatment effect rate of the two groups.

Group	Hips	Excellent	Good	General	Poor	Excellent & good rate
PRP & simvastatin	77	15 (19.5)	51 (66.2)	4 (5.2)	7 (9.1)	66 (85.7)
Simvastatin	80	5 (6.3)	52 (65.0)	7 (8.7)	16 (20.0)	57 (71.3)
*χ^2^*						4.838
*P*						0.028

**Fig.2:**
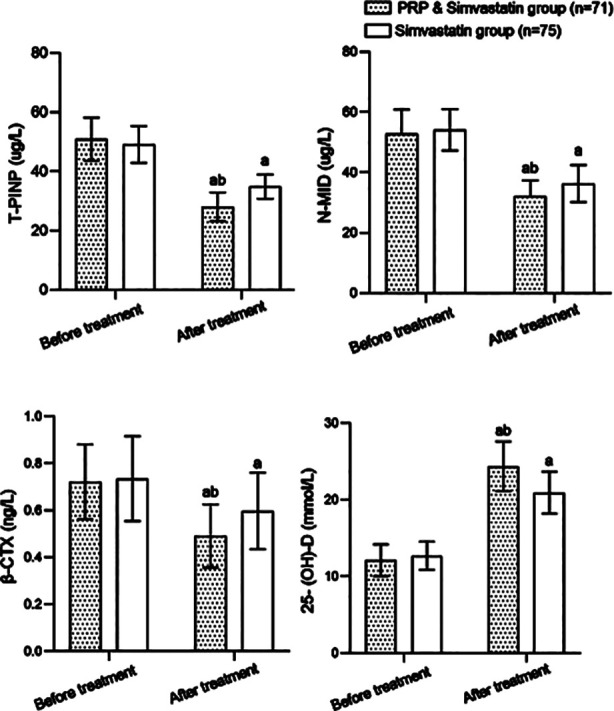
The histogram of bone metabolism index changes in the two groups before and after treatment; Compared with before treatment in the same group. ^a^P<0.05; compared with simvastatin group, ^b^P<0.05; PRP: Platelet rich plasma; T-PINP: total-Type-I collagen N-terminal propeptide; N-MID: N-terminal molecular fragment; β-CTX: β-isomerized C-terminal telopeptide of Type-I collagen; 25-(OH)-D: 25 hydroxyvitamin D.

No significant difference was found in the levels of T-PINP, N-MID, β-CTX and 25- (OH) -d between the two groups before treatment (*P*>0.05). The levels of T-PINP, N-MID, and β-CTX in both groups considerably decreased after treatment and were significantly lower in patients who were treated by PRP combined with simvastatin compared to patients who received simvastatin alone. The combined treatment was associated with significantly higher post-treatment levels of 25-(OH)-D compared to the simvastatin alone (*P*<0.05) (Fig.2). No significant difference was found in the incidence of ARs between the PRP plus simvastatin group (9.9%) and the simvastatin group (4.0%) (*P*>0.05) (Table-III).

**Table-III:** Comparison of the incidence of ARs between the two groups.

Group	n	Abdominal pain	Abdominal distension	Vomiting and nausea	Headache	Total incidence
PRP & simvastatin	71	3 (1.3)	2 (1.3)	0 (0.0)	2 (2.6)	7 (9.9)
simvastatin	75	1 (1.4)	1 (1.3)	1 (1.4)	0 (0.0)	3 (4.0)
*χ^2^(corrected)*						1.152
*P*						0.283

## DISCUSSION

This study showed that compared with simvastatin alone, the combination of PRP and simvastatin was safe and more efficient in treating SANFH than simvastatin alone. Numerous studies have showed that long-term use of corticosteroids can lead to abnormal secretion of vasoactive substances and changes in vascular permeability around the femoral head,^3^,^4^,^14^ negatively affecting vascular endothelial cells and leading to microthrombosis, slower blood flow velocity, and damage to the normal tissue structure around the femoral head.^4^,^15^

Several studies have confirmed the application value of simvastatin or PRP alone in treating osteonecrosis of the femoral head (ONFH).^16^,^17^ Yin H et al.^16^ showed that simvastatin treatment is associated with an improved therapeutic effect and reduced risk of femoral head collapse. Tong SC et al.^17^ showed that PRP can promote beneficial effects by preventing joint inflammation, cartilage, and bone damage and stimulating joint tissue repair in mice with ONFH. A study by Luan S et al.^18^ confirmed that intra-articular injection of PRP significantly improved the pain scores and HHS of ONFH patients with no reported adverse events. The findings of the present study align with these conclusions. The beneficial effect of PRP treatment may be related to a high content of various growth factors that can promote angiogenesis and improve the blood supply of the femoral head.

Moreover, studies have showed that in ONFH patients, PRP could inhibit the activity of osteoclasts, promote differentiation and proliferation of osteoclasts, reduce bone resorption, and promote bone repair.^19^,^20^ This study aimed to assess the impact of the comprehensive application of simvastatin and PRP on SANFH to clarify the benefits of combining the two drugs in treating this condition. Our results showed that the combined treatment led to better outcomes in terms of joint activity, function, and pain scores. Moreover, the rate of excellent and good treatment effects (85.7%) of the combined regimen was significantly higher than that of simvastatin alone (71.3%) after treatment. The incidence of ARs was comparable in both groups. Our results further confirm previous reports that combining simvastatin and PRP in treating SANFH is safe and can improve joint function and the treatment effect.^21^,^22^

It has been found that long-term use of corticosteroids can reduce the blood supply of the surrounding tissues of the femoral shaft, hinder the blood supply of the surrounding normal tissues of the femoral head, activate the activity of osteoclasts, and inhibit the activity of osteoblasts.^23^,^24^ T-PINP, N-MID, β-CTX and 25-(OH)-D are common indicators of bone metabolism assessment.^25^,^26^ This study found that PRP combined with simvastatin had a more significant effect on T-PINP, N-MID, and β-CTX and 25-(OH)-D levels compared to simvastatin alone. We may speculate that the observed differences in the inflammatory markers may be due to the ability of PRP to regulate the function of the immune system and reduce inflammatory reactions, as was demonstrated by previous research.^19^,^20^

### Strengths:

The main strength of this study is its novelty, as currently, there are no reports to confirm the clinical value of combining simvastatin and PRP in the treatment of SANFH. Therefore, this study can fill the gap in related fields and provide new ideas for the clinical treatment of diseases. However, the sample size of this study is small, and high-quality trials are still needed to verify the therapeutic effect of PRP combined with simvastatin.

### Limitations:

Firstly, it is a retrospective single-center study with a small sample size. Future large-sample studies with specific patient cohorts are needed to confirm our results. Additionally, in this study, patients who completed four weeks of treatment without confirmed disease progression were allowed to continue to use PRP, since, according to the investigator’s assessment, they were considered to benefit from the study treatment. Finally, large-scale controlled studies, including patients with other causes of femoral head necrosis, are needed to verify the benefits of PRP combined with simvastatin.

## CONCLUSION

Compared with simvastatin alone, the combination of PRP and simvastatin in treating SANFH can more effectively restore the patient’s joint function, regulate bone metabolism, improve the treatment efficiency, and is not associated with increased incidence of adverse effects.

### Author’s contributions:

**YC:** Study design, literature search and manuscript writing.

**CR, HQ, HZ and YF:** Data collection, data analysis and interpretation. Critical Review.

**YC:** Manuscript revision and validation, Critical Analysis.

All authors have read, approved the final manuscript and are responsible for the integrity of the study.
